# Functionalized Superparamagnetic Iron Oxide Nanoparticles as a Sustainable Approach for Gas Hydrate Control

**DOI:** 10.1021/acsomega.5c01960

**Published:** 2025-05-22

**Authors:** Ali H. Karaly, Malcolm A. Kelland, Mohamed F. Mady

**Affiliations:** † Department of Chemistry, Bioscience and Environmental Engineering, Faculty of Science and Technology, 56627 University of Stavanger, Stavanger N-4036, Norway; ‡ Department of Chemistry and Earth Sciences, College of Arts and Sciences, 61780 Qatar University, 2713 Doha, Qatar

## Abstract

Gas hydrate blockage in multiphase flow lines is a critical issue in upstream operations. One method to prevent this from happening is by the use of kinetic hydrate inhibitors (KHIs). KHIs are polymers containing tailored amphiphilic groups. These polymers often have limited marine biodegradability, and their recovery and recycling to reduce operational costs remain a challenge. A novel approach involves attaching KHIs to magnetic nanoparticles, enabling recovery and recycling without environmental discharge. We have developed superparamagnetic iron oxide nanoparticles (SPIONs) reacted first with vinyltrimethoxysilane (VTMS) and then coated with *N*-vinylpyrrolidone/*N*-vinyl caprolactam (VP/VCap) copolymer chains using radical polymerization of the VP and VCap monomers (SPIONs-VTMS-VPVCap). These nanoparticles are stable in aqueous solutions with a particle size of 10 nm and a dispersion size of 205 nm. High-pressure tests demonstrated that SPIONs-VTMS-VPVCap performed comparably to the free VP/VCap copolymer, achieving a hydrate formation onset temperature (*T*_o_) of 12.9 °C at 5000 ppm. Significantly, the magnetic KHIs were successfully recovered and reused multiple times without performance loss. The solution exhibited a high cloud point (80 °C) and compatibility with *n*-butyl glycol ether (BGE), enhancing the performance. Adding 5000 ppm of BGE lowered the hydrate formation *T*_o_ to 7.3 °C, a 9.7 °C improvement compared to no additive. These results establish a proof of concept for recyclable magnetic KHIs, offering a sustainable solution to eliminate chemical discharge in marine environments while maintaining effective hydrate inhibition.

## Graphical abstract

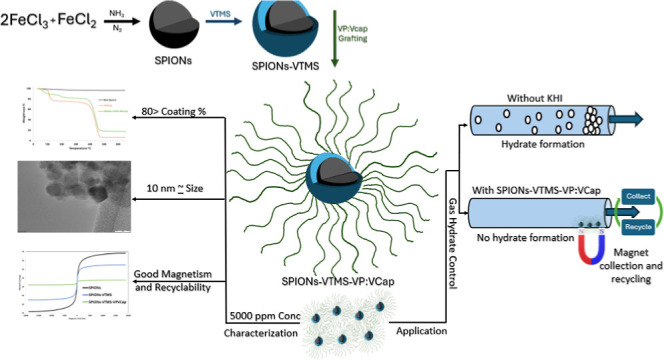

## Introduction

The upstream oil and gas industry faces many challenges with regards to polluting the environment including the land, sea, and air.^1^ Many oilfield chemicals follow the aqueous phase in the produced fluids, either partially or the whole dosage. Examples include scale, corrosion, and gas hydrate inhibitors.^2,3^ Governmental authorities have implemented strategies to protect the marine environment from harmful chemicals discharged from offshore platforms. These measures can include reducing the level of chemicals, replacing potentially harmful chemicals with more benign chemicals, and recycling chemicals. Some authorities are even pushing for zero discharge, as the long-term chronic effects of many chemicals are not known.

In the last two decades, nanotechnology has provided many solutions to problems within the petroleum industry.^4–12^ However, there is some concern regarding the fate of nanoparticles that are released into the marine environment.^13^ One way to avoid complete chemical discharge when nanoparticles are used is to deploy magnetic nanoparticles (MNPs) that can be recycled. In short, the active chemical is bound to the MNPs, injected where needed into the system to do its job, recovered using magnets, and reinjected again. In this way, theoretically, there is zero chemical discharge into the environment. A typical scenario where MNPs could be used is in multiphase production flow lines. Several groups including our own have developed MNPs for scale inhibition.^14–17^

Attempts have also been made to make MNPs for preventing gas hydrates from plugging flow lines. These are discussed below after a short introduction to gas hydrates and the various chemical inhibition methods available. Gas hydrates are clathrates, ice-like solids formed from natural gases and water at high pressure and temperatures below about 25 °C.^18–20^ The commonest hydrate inhibitors are thermodynamic inhibitors (THIs) but they require high concentrations and therefore large volumes to be effective. Therefore, they are not appropriate for functionalizing onto MNPs. Low-dosage hydrate inhibitors (LDHIs) are deployed at <1–3 wt % of the aqueous phase and are more amenable to being attached to MNPs.^21^ Thus, although the MNPs coated with LDHI will be more expensive than the LDHI alone, the ability to recycle the MNPs will drastically reduce operational costs.

There are two main classes of LDHIs, antiagglomerants (AAs) and kinetic hydrate inhibitors (KHIs). AAs are surfactants and interfere with the crystal growth and agglomeration stages of hydrate formation such that a flowable, nondepositing dispersion of hydrate particles is formed. Most commercial AAs are quaternary ammonium surfactants.^22^ KHIs delay the formation of gas hydrates inside the thermodynamic region for hydrate formation.^23^ The delay time is related to the driving force (chemical potential of the system) to form hydrates. The subcooling (the equilibrium temperature minus the system temperature) is often used to define the driving force for hydrate formation. KHIs have multiple amphiphilic groups, usually oligomers or polymers. Commercial KHIs are exclusively polyamide-based. Solvents, which can act as synergists, are added to boost the performance. A common example is *n*-butyl glycol ether (BGE).^24^ Commercial examples include polymers and copolymers of the vinyl lactam monomers *N*-vinyl caprolactam (VCap) and *N*-vinylpyrrolidone (VP) and *N*-isopropylmethacrylamide (NIPMAm) (Figure 1).^25–33^

**Figure 1:**
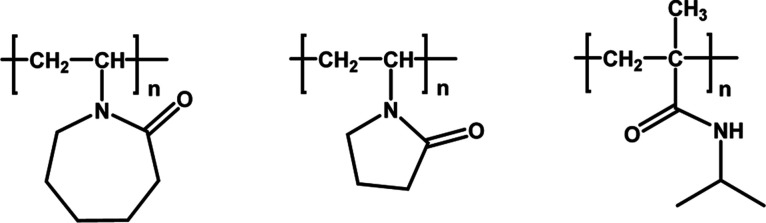
Left to right, poly­(*N*-vinyl caprolactam) (PVCap), poly­(*N*-vinylpyrrolidone) (PVP), and poly­(*N*-isopropylmethacrylamide) (PNIPMAm).

KHI polymers often have cloud points in an aqueous medium. The cloud point is the temperature above which phase separation of the polymer occurs from the solution. This can lead to fouling, unwanted deposition of the polymer if the system temperature is above the cloud point.^34^ Homopolymers of VCap or NIPMAm as 0.1–1.0 wt % aqueous solutions have low cloud points of about 30–40 °C in pure water. These values are even lower in brines. Therefore, these homopolymers are rarely deployed as KHIs as the system temperature, such as the wellhead injection point, is often above the polymer cloud point. Therefore, the most used KHI polymers containing VCap or NIPMAm are copolymers with more hydrophilic monomers. An example is VPVCap copolymer.^23,27,33^

MNPs coated with Span 20 surfactant have been reported as potential AA.^35^ While recoverability was demonstrated through vibrating-sample magnetometer (VSM) data, the recyclability of the nanocomposite was not explored. Furthermore, it remains unclear whether the Span 20 coating persists on the MNPs after hydrate formation. The efficiency of the AA also showed high sensitivity to the ratio of Span 20 to MNPs, which may limit its scalability for larger systems. Amine-functionalized metal–organic frameworks (MOFs) have also been studied as KHIs.^36^ The reported performance was relatively modest, and direct evidence confirming the presence of primary amine (−NH_2_) functionalization through techniques such as Fourier-transform infrared spectroscopy (FTIR) was not provided.

Nanocomposites involving poly­(vinylcaprolactam) (PVCap) and poly­(vinylpyrrolidone) (PVP) coatings on γ-methacryloxypropyltrimethoxysilane (MPS)-modified Fe_3_O_4_ nanoparticles have shown potential as KHIs.^37,38^ However, challenges were observed when attempting to replicate the synthesis process related to the stoichiometry, solubility, efficiency, and overall applicability in our own laboratory. Nanoparticle organic hybrid materials (NOHMs), such as NOHM-I-PNIPAM, have demonstrated interesting properties, including a tunable Fe_3_O_4_ core-SiO_2_ shell structure with poly­(*N*-isopropylacrylamide) (PNIPAM).^39^ These nanocomposites reduced hydrate onset temperatures by 4.39 °C at a 1 wt % dosage during controlled cooling tests. However, the long-term dispersibility and stability of the nanoparticles in aqueous solutions were not assessed. Although magnetic recoverability was demonstrated, further studies are needed to evaluate the material’s reusability and performance consistency after recovery.

Here, we present results of the synthesis, characterization, stability, KHI performance, and recycling of MNPs (Fe_3_O_4_) coated with high cloud point *N*-vinyl lactam chains. The MNPs contain up to 80% by weight KHI-active polymer chains. These MNPs show good KHI performance, excellent long-term dispersion stability and stable performance in multiple tests after recycling with a magnet. These features are necessary for deploying KHIs where long residence times are needed in the flow line, such as long tiebacks or in the case of potential shut-in/restart scenarios. The MNPs are also compatible with *n*-butyl glycol ether solvent which also boosts the performance significantly.

## Experimental Section

### Materials

All chemicals and solvents used in this study were of analytical grade and were obtained from Sigma-Aldrich (Merck), VWR Chemicals, and Tokyo Chemical Industry Co., Ltd., unless otherwise stated. Iron­(III) chloride hexahydrate (FeCl_3_·6H_2_O) and iron­(II) chloride tetrahydrate (FeCl_2_·4H_2_O) were employed as the sources of Fe^3+^ and Fe^2+^ for the coprecipitation synthesis of superparamagnetic iron oxide nanoparticles (SPIONs). Ammonium hydroxide (25% NH_4_OH) was used as the alkaline precipitant. Vinyltrimethoxysilane (VTMS) was selected as the silica coating precursor due to its vinyl functional group. Vinylpyrrolidone (VP, 99%) and vinyl caprolactam (VCap, 99%) were used as monomers in free radical polymerization to functionalize the SPIONs. Azobis­(isobutyronitrile) (AIBN) served as the radical initiator for the polymerization reaction. Ethanol (C_2_H_5_OH), isopropanol (IPA), tetrahydrofuran (THF), and *n*-hexane were used as solvents during the synthesis and purification processes. Deionized (DI) water was used for all solution preparations and nanoparticle washing.

The instruments used in the study included a Z671797 IKA C-MAG HS hot plate stirrer, a pH/mV/°C meter (pHenomenal pH 1100 L, VWR), and a PC 3001 VARIOpro EK Peltronic rotary evaporator with an RV 10 digital pro V Complete IKA. Ultrasonic processes were carried out using a Branson Sonifier Digital Ultrasonic Cell Disruptor and a Bransonic ultrasonic cleaner. Milli-Q water, used throughout the experiments, was sourced from an ELGA PURELAB Option-R 7 system. Magnetic separation was employed for collecting nanoparticles using a neodymium magnet.

### Synthesis of Superparamagnetic Iron Oxide Nanoparticles

SPIONs were synthesized via the coprecipitation of Fe^2+^ and Fe^3+^ ions in an alkaline medium. Briefly, 1.26 g of FeCl_2_·4H_2_O (1 equiv of Fe^2+^) and 3.5 g of FeCl_3_·6H_2_O (2 equiv of Fe^3+^) were dissolved in 400 mL of deoxygenated deionized (DI) water. The solution was continuously bubbled with nitrogen in a two-necked Erlenmeyer flask fitted with a thermometer and a funnel. Under nitrogen flow, an excess of ammonium hydroxide solution (12 g of 25% NH_4_OH) was added dropwise, and the mixture was stirred vigorously for 1 h. The solution was then heated to 70 °C for 30 min to evaporate any unreacted NH_4_OH. The resulting black precipitate was washed three times with DI water and two times with ethanol to remove excess water, before magnetic separation was used to collect the SPIONs for subsequent steps. SPION black powder weighed 1.45 g.

### Synthesis of SPIONs-VTMS

For surface functionalization, SPIONs were coated with vinyltrimethoxysilane (VTMS) via basic hydrolysis. 3 mL of VTMS was used as both a precursor for silica coating and a linker, due to its vinyl functional group. The nanoparticles from the previous step were dispersed in a solution of 240 mL of ethanol, 10 mL of DI water, and 12 mL of NH_4_OH. This mixture was sonicated for 20 min before the dropwise addition of VTMS. The reaction mixture was sonicated for an additional 10 min and stirred at 50 °C for 48 h. The VTMS-coated SPIONs were washed with DI water and recollected by magnetic separation until the pH reached 7. The coated nanoparticles were dried under reduced pressure using a rotary evaporator and weighed 1.87 g.

### Synthesis of SPIONs-VTMS-VPVCap

A vinylpyrrolidone (VP) and vinyl caprolactam (VCap) copolymer layer was grafted onto SPIONs-VTMS via free radical polymerization. First, 1 g of SPIONs-VTMS was suspended in a solution of 8.33 g of VP (75 mmol) and 10.44 g of VCap (75 mmol) in 250 mL of isopropanol (IPA). After sonication for 10 min, the solution was degassed and bubbled with nitrogen for five cycles. Then, 0.2 g of azobis­(isobutyronitrile) (AIBN) was added to initiate polymerization at 80 °C for 24 h. The solvent was removed by rotary evaporation, and the residue was redispersed in 200 mL of tetrahydrofuran (THF). *n*-Hexane (70 mL) was added to aid the collection of SPIONs-VTMS-VPVCap using magnetic separation, as VPVCap chains get reformed in *n*-hexane, which was repeated five times to ensure thorough washing of the nanoparticles. Finally, the SPIONs-VTMS-VPVCap were dried using rotavap and weighed 12.21 g (Scheme 1).

**Scheme 1:**
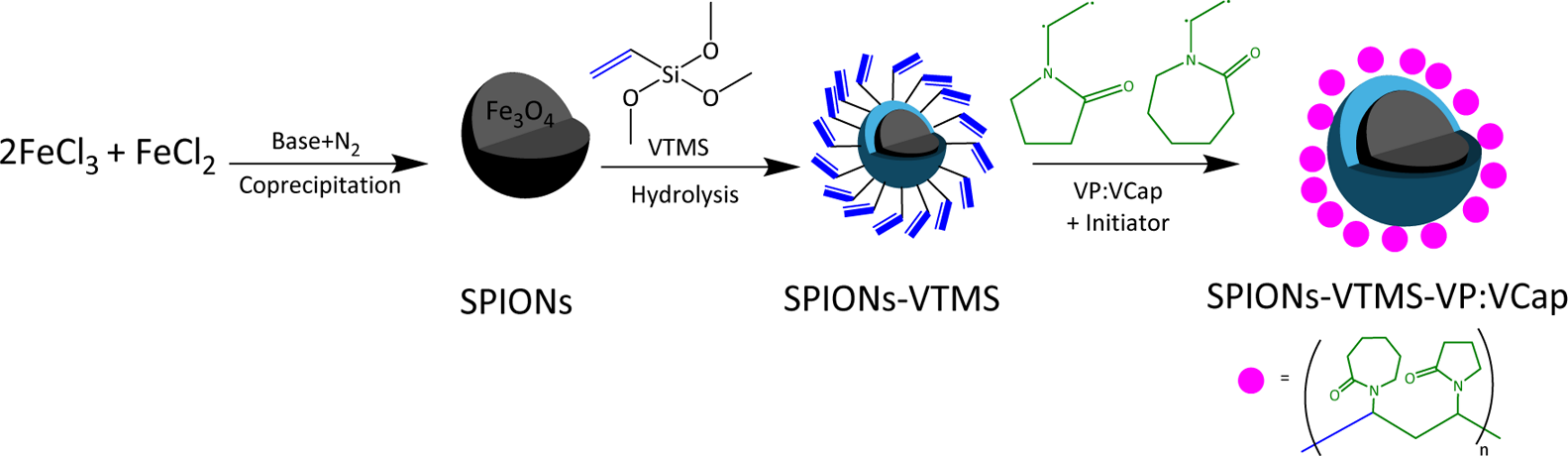
Synthesis of SPIONs-VTMS-VPVCap as a KHI

### Synthesis of the VPVCap Copolymer

For comparison, a freestanding VPVCap copolymer was synthesized using the same method as above but without SPIONs. Specifically, 0.75 g of VTMS (5.25 mmol) was dissolved in a solution containing 8.33 g of VP (75 mmol) and 10.44 g of VCap (75 mmol) in 250 mL of isopropanol (IPA). The solution was degassed, and nitrogen was bubbled through for five cycles, followed by the addition of 0.2 g of AIBN to initiate the reaction at 80 °C for 24 h. After the reaction, the solvent was removed by rotary evaporation, and the residue was collected and used without further purification.

### Characterization

Thermogravimetric analysis (TGA) was carried out with a Mettler Toledo TGA/DSC 3+ system under a nitrogen atmosphere (25 mL/min), with heating from 25 to 600 °C at a rate of 10 °C/min. The morphology and particle size were examined by transmission electron microscopy (TEM) using a JEM-2100 Plus instrument operating at 200 kV with a 0.19 nm spatial resolution (LaB_6_ electron gun). Dynamic light scattering (DLS) and zeta potential measurements were performed with a Malvern Zetasizer Nano series, using DI water (pH 7) as the dispersing medium. The crystallinity of the synthesized nanoparticles was determined using X-ray diffraction (XRD) analysis on a Bruker D8 Advance diffractometer, operating at 40 kV and 40 mA, with a 2θ range of 10°–80° and a Cu Kα source (λ = 1.5406 Å). Prior to measurement, the particles were sonicated for 5 min, and results were averaged over 10 readings. Fourier-transform infrared (FTIR) spectra were collected using an Agilent Cary 630 FT-IR spectrophotometer equipped with an attenuated total reflectance (ATR) attachment, with measurements taken between 400 and 4000 cm^–1^. ^1^H nuclear magnetic resonance (NMR) spectroscopy was performed to confirm the chemical structure of the synthesized polymer. The ^1^H NMR spectra were recorded on a Bruker 400 MHz spectrometer using deuterium oxide (D_2_O) as the solvent. The VPVCap sample was dissolved in D_2_O, and chemical shifts (δ) were reported in parts per million (ppm). Magnetic properties were evaluated at 300 K using a quantum design MPMS instrument (SQUID VSM).

### Kinetic Hydrate Inhibitor Performance Tests

The KHI performance of the synthesized polymers was evaluated using a high-pressure rocking cell rig supplied by PSL Systemtechnik, Germany (Figure 2). The cells were constructed by Swafas, Norway. This rig consisted of five parallel high-pressure stainless-steel cells in a thermally controlled water bath. The system was operated using a synthetic natural gas (SNG) blend composed of methane (80.67%), ethane (10.2%), propane (4.90%), iso-butane (1.53%), *n*-butane (0.76%), carbon dioxide (1.84%), and dinitrogen (0.10%) made by Yara Praxair, Norway. The equilibrium temperature (Teq) for structure II gas hydrates at 76 bar of SNG was determined to be 20.5 °C using the PVTSim software (Calsep).

**Figure 2:**
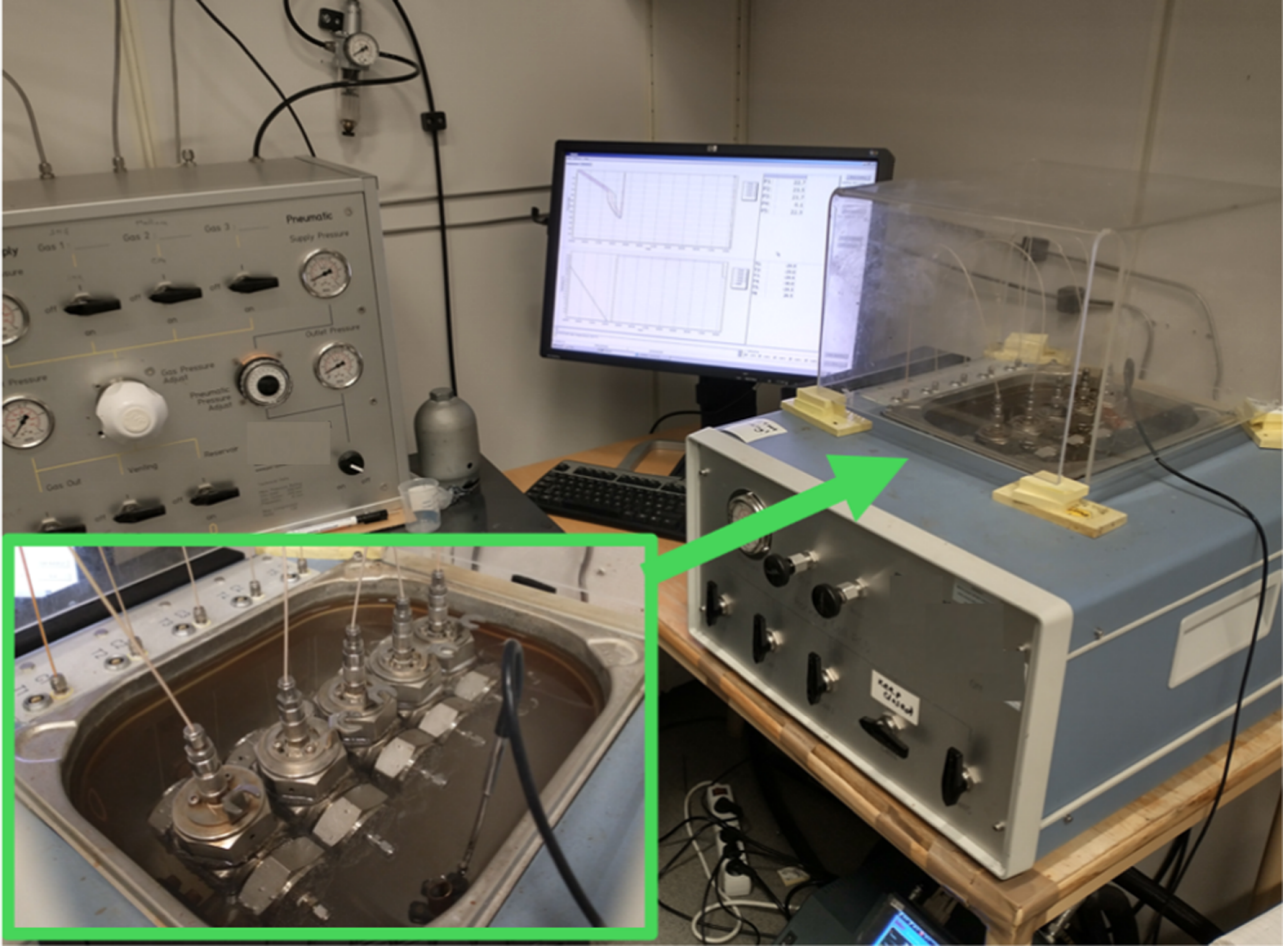
High-pressure KHI multiple rocking cell test equipment.

The kinetic hydrate inhibition test procedure has been described before.^40,41^ For a set of five rocking cell tests, each sample was dissolved in 105 mL of deionized water and prepared 24 h before the experiment. Twenty milliliters of this solution was injected into each cell and sealed tightly. After two cycles of purging the cells with SNG followed by vacuum to remove any residual air, the cells were loaded with 76 bar of SNG at 20.5 °C and individually shut down at the gas inlet/outlet valves. The rocking cells were rocked at 20 rocks/min and slowly cooled at 1 °C per hour, while sensors continuously monitored pressure and temperature.

The slow constant cooling (SCC) method was employed to evaluate KHI performance as the cells were slowly cooled from pressure 76 bar and temperature 20.5 to 2 °C at a controlled rate of 1 °C per hour, a technique designed to simulate real field conditions. The slow cooling allows for accurately detecting the onset of hydrate formation and helps mimic the gradual temperature drops seen in offshore pipelines.^42,43^ Additionally, the cooling rate of 1 °C per hour is established to give a test that can be conducted in 24 h yet is long enough to discern differences in performance.

The hydrate onset temperature (*T*o) was determined as the temperature at which the first deviation in pressure occurs from the linear decrease curve. An example is shown in Figure 3. Microscopic hydrate nucleation may have occurred somewhat earlier than *T*_o_ but *T*_o_ is still a good value to compare the relative performance of KHIs. The *T*_o_ value is considered the most important parameter, as operators want to completely prevent hydrate formation and potential deposition in their flowlines. The rapid hydrate formation temperature (*T*_a_) was also recorded, representing the temperature at which the hydrate growth rate first reaches its maximum value. The *T*_o_–*T*_a_ value can give some idea of the ability of a KHI to arrest hydrate crystal growth. However, caution must be used, as growth rates for two different KHIs are only comparable if the driving force (subcooling) is similar in both experiments. The *T*_o_–*T*_a_ value can be helpful to the operator because a KHI giving a slow growth rate will give the operator time to react with remedial treatment before the flow line is plugged with hydrates. Each polymer was tested in five parallel experiments, and the *T*_o_ and *T*_a_ values were averaged. Statistical significance between different polymer samples was assessed using the *t*-test, with a *p*-value of less than 0.05, indicating a significant difference in KHI performance. The variation within the *T*_o_ values is usually ±10–15% and about ±10% for the *T*_a_ values. A typical set of full values and their interpretation has been reported.^44^

**Figure 3:**
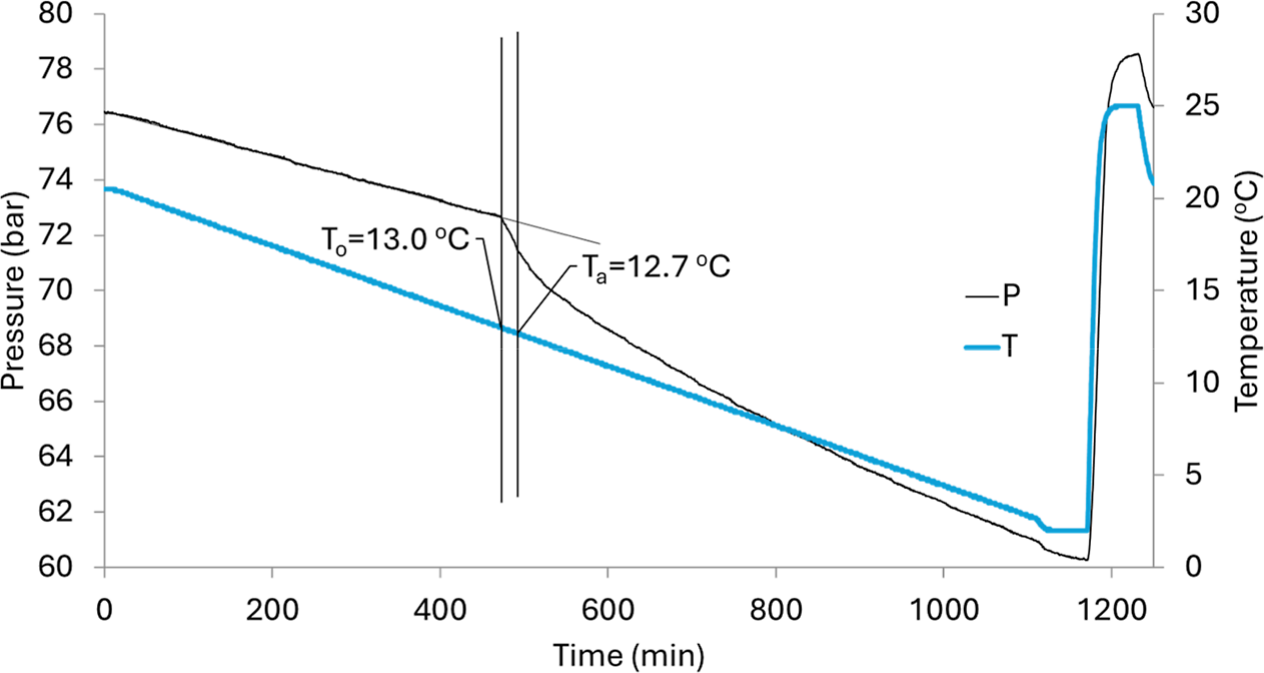
SCC test result from a rocking cell containing 5000 ppm of SPIONs-VTMS-VPVCap. The temperature *T* is taken from the cooling bath.

## Results and Discussion

### Thermogravimetric Analysis

Thermogravimetric analysis (TGA) was performed to assess the synthesized materials’ thermal stability, polymeric content, and weight loss. The TGA curves for bare SPIONs, VPVCap, and SPIONs-VTMS-VPVCap are depicted in Figure 4, with a temperature range of 25–650 °C. The analysis revealed that the bare SPIONs exhibited minimal weight loss of approximately 3%, indicating their stability under thermal stress. In contrast, the VPVCap demonstrated significant thermal degradation with a weight loss of around 92%, which suggests a substantial loss of the polymeric structure at elevated temperatures. The SPIONs-VTMS-VPVCap showed a weight loss of approximately 85%, reflecting its high polymeric content and success in grafting the VPVCap copolymer onto the VTMS linker. The initial weight loss observed at around 100 °C for all samples is attributed to the evaporation of moisture, while the second pronounced weight loss at around 450 °C corresponds to the degradation of the VPVCap polymer. These results indicate the successful functionalization of SPIONs with VTMS and VPVCap with more than 80% polymeric layer.

**Figure 4:**
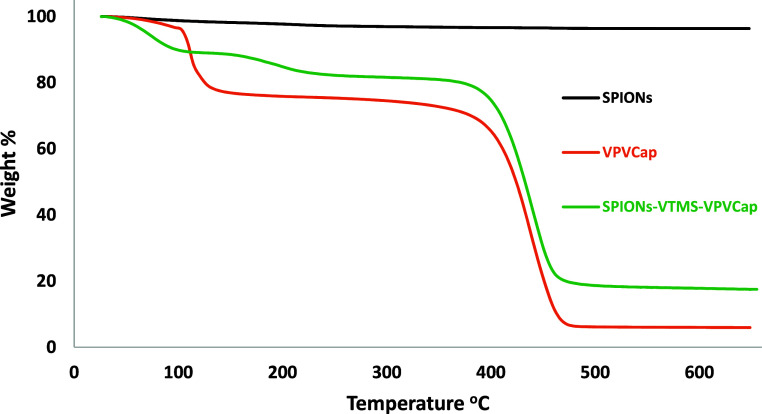
TGA curves of SPIONs, VPVCap polymer, and SPIONs-VTMS-VPVCap polymer.

### Transmission Electron Microscopy

The TEM image reveals the morphology of the synthesized magnetic nanoparticles, showing them to be quasi-spherical in shape with a tendency to aggregate, likely due to high surface tension in the dry state, as shown in Figure 5. In each of the images labeled (a)-(d), we see distinct clusters of nanoparticles, indicating a relatively uniform particle size distribution. The histogram in image (a) suggests a narrow size distribution, indicative of monodispersity.

**Figure 5:**
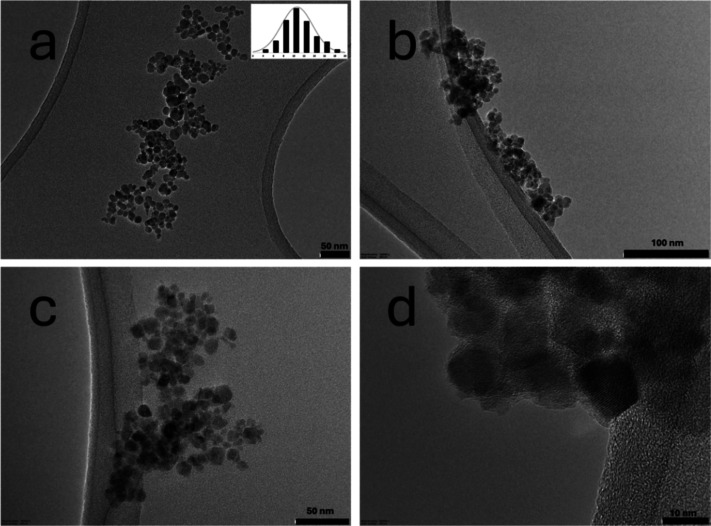
TEM image of (a) SPIONs at 50 nm scale, (b) SPIONs-VTMS at 100 nm scale, (c) SPIONs-VTMS-VPVCap at 50 nm scale, and (d) SPIONsVTMS-VPVCap at 10 nm scale.

SPIONs-VTMS-VPVCap (d) appear to be around 10.5 nm in diameter, consistent with the bare SPIONs histogram (a). In these images, the core structure is visible, and the particles are fairly uniform in shape and size. The laminar pattern visible in image (d) suggests well-defined crystalline planes in the SPIONs-VTMS core, while the surrounding areas are more amorphous, possibly indicating the polymer coating. Although no clear polymer layer can be observed here, the consistency of particle shape and lack of aggregation suggest a successful coating without altering the primary particle characteristics.

This image series supports the idea that the coating process does not affect the main morphological characteristics of the particles, confirming that the particles retain their quasi-spherical shape and monodisperse size distribution. The high-resolution image in (d) highlights the crystalline nature of the SPIONs-VTMS core, reinforcing that the core remained intact and unchanged during coating.

### Dynamic Size and Zeta Potential

The zeta potential distribution shows a sharp peak centered around zero, which indicates a neutral surface charge, as shown in Figure 6a. This neutral charge is due to the nature of the VPVCap polymer coating on the SPIONs, confirming the successful polymer attachment. The polymer coating neutralizes the surface charge, resulting in a near-zero zeta potential, reflecting the stability of steric (rather than electrostatic) stabilization.^45^

**Figure 6:**
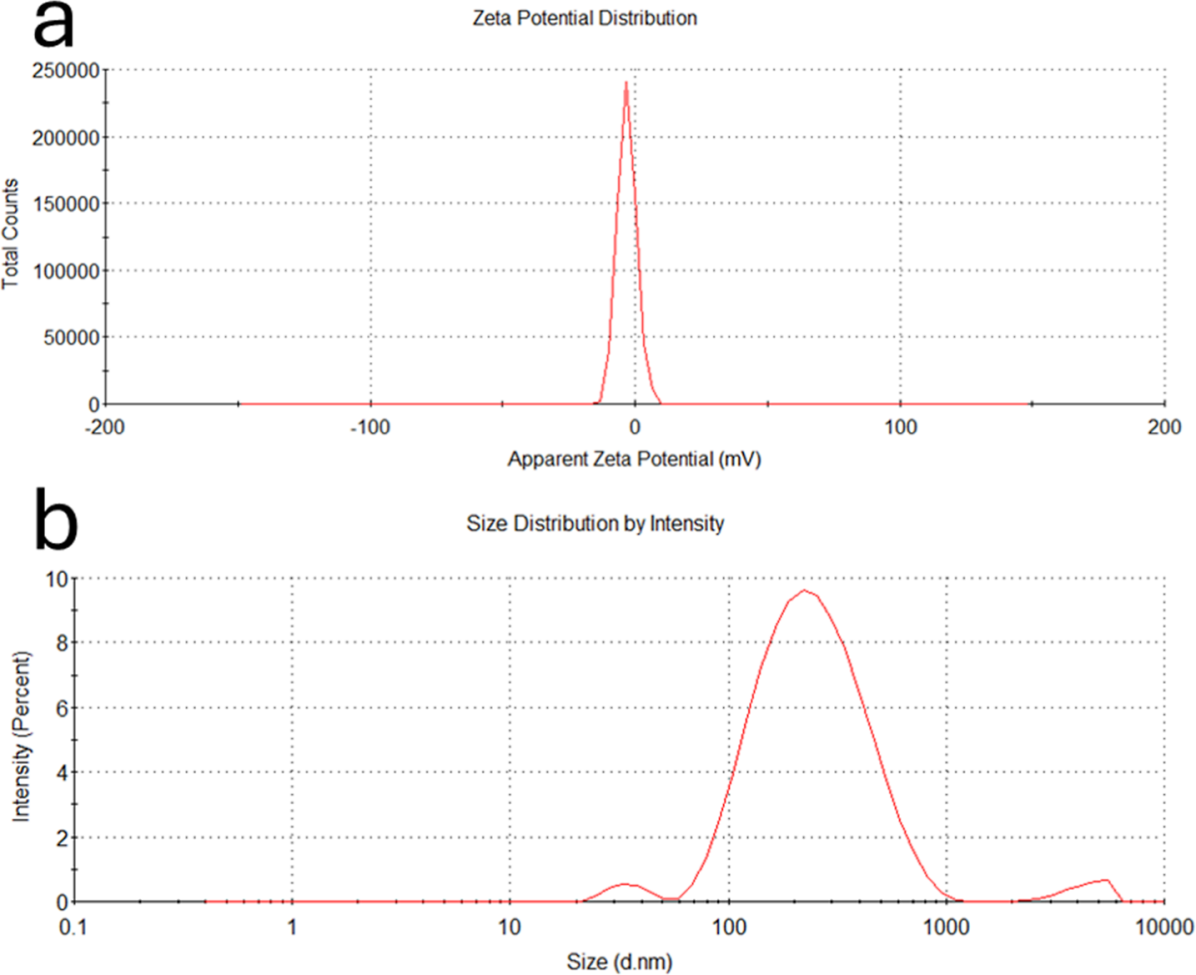
DLS-zeta curves of SPIONs-VTMS-VPVCap. (a) Zeta potential of SPIONs-VTMS-VPVCap; (b) DLS analysis of SPIONs-VTMS-VPVCap.

DLS analysis shows a relatively narrow peak with a polydispersity index (PDI) of 0.27 and an average particle size of approximately 205 nm, as shown in Figure 6b. This narrow distribution and low PDI indicate a stable and uniform particle size without significant aggregation, which aligns with successful coating and dispersion in solution. The moderate particle size (205 nm) suggests that the polymer coating has effectively prevented substantial aggregation, maintaining the SPIONs as discrete particles in suspension.

Overall, these results suggest that the polymer coating on the SPIONs enhances stability through steric effects, as evidenced by the near-zero zeta potential and relatively low PDI. This coating ensures that the particles remain well-dispersed and minimizes their tendency to aggregate, supporting the effectiveness of the coating for stabilizing SPIONs in suspension.

### X-ray Diffraction

X-ray diffraction (XRD) analysis was employed to investigate the crystalline structure of the synthesized superparamagnetic iron oxide nanoparticles (SPIONs) and to confirm the formation of the desired magnetite phase (Figure 7). The XRD patterns were recorded in the 2θ range of 10°–80° using a Bruker D8 Advance diffractometer with Cu Kα radiation. The prominent peaks obtained for SPIONs were located at 2θ values of 18.5°, 30.2°, 35.5°, 43.4°, 53.8°, 57.2°, 62.9°, 66.0°, and 74.5°. These peaks can be assigned to the magnetite planes (1 1 0), (2 2 0), (3 1 1), (2 2 2), (4 0 0), (4 2 2), (5 1 1), (4 4 0), (5 3 1), and (6 2 2), respectively, in accordance with the RRUFF ID: R061111. The sharpness and intensity of these peaks indicated a high degree of crystallinity in the synthesized SPIONs.^46^

**Figure 7:**
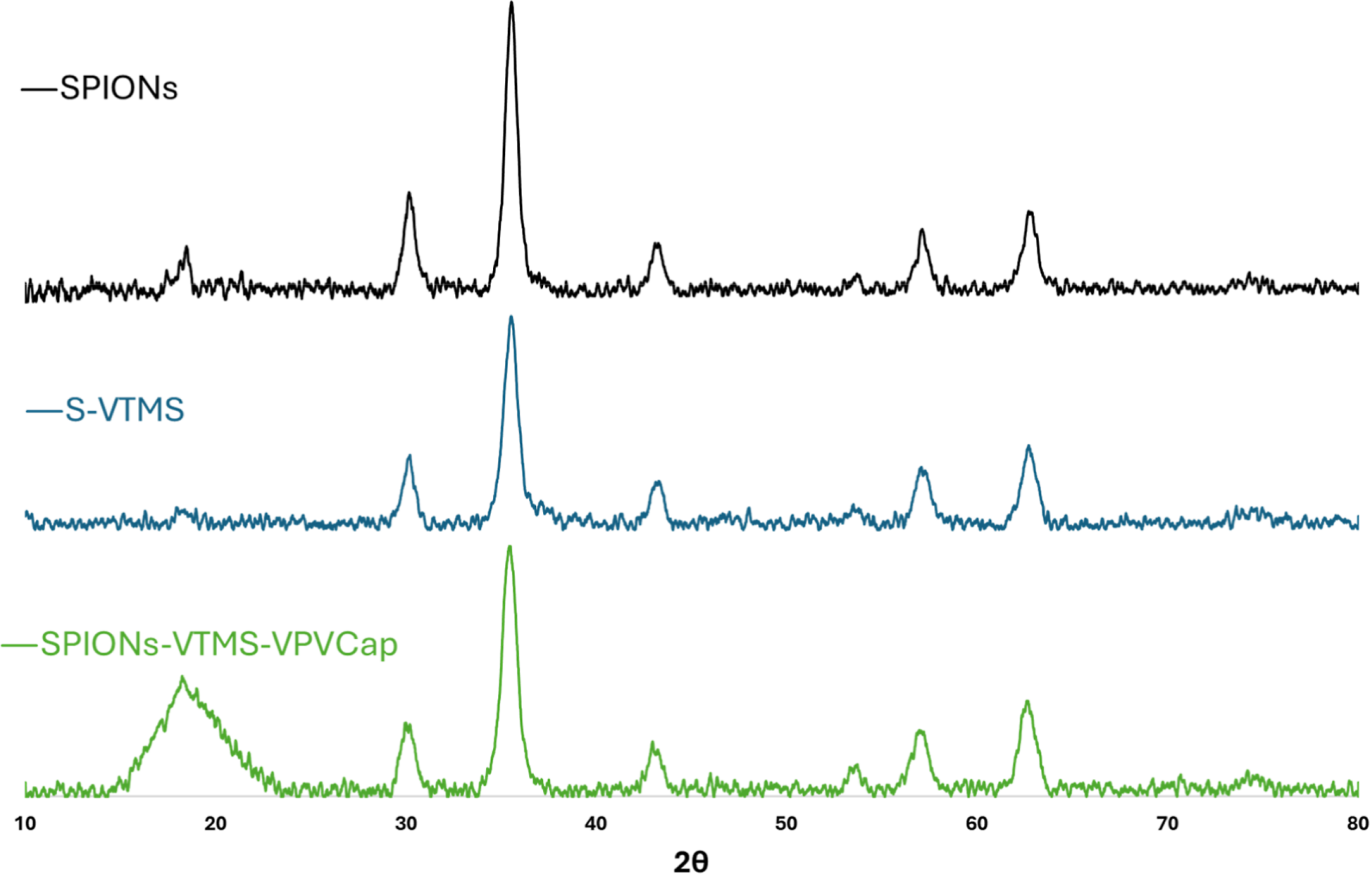
XRD patterns of SPIONs, SPIONs-VTMS, and SPIONs-VTMS-VPVCap.

For the SPIONs-VTMS nanoparticles, weak broad peaks in the range of 2θ = 17°–19° were detected, suggesting the presence of amorphous SiO_2_, which is consistent with the silica coating introduced during the functionalization process.^47^ Additionally, for SPIONs-VTMS-VPVCap, a relatively broad peak centered at 2θ equal to 18.4° was observed, indicating the amorphous nature of the polymer component.^48^ This finding suggests that while the SPIONs maintain their crystalline characteristics, the polymer and silica do not exhibit a defined crystalline structure, further confirming the successful incorporation of these materials onto the surfaces of the nanoparticles.

### Fourier Transform Infrared Spectroscopy

Fourier transform infrared (FTIR) spectroscopy was performed to investigate the functional groups present on the surfaces of the synthesized nanoparticles. The FTIR spectra of SPIONs-VTMS samples showed distinct peaks over SPIONs, with the most prominent one at 1088 cm^–1^, corresponding to the Si–O–Si asymmetric stretching vibrations, which are characteristic of the silica component in the VTMS-functionalized nanoparticles.^49^ Additionally, the presence of vinyl groups was confirmed by the appearance of bands at 1407 cm^–1^ and 1600 cm^–1^, attributed to CH asymmetric bending vibrations and CH_2_ stretching vibrations on Si—CH=CH_2_, respectively.^50,51^ These bands are indicative of the successful grafting of VTMS onto the surface of the SPIONs.

For the SPIONs-VTMS-VPVCap samples, the FTIR spectra displayed a broad absorption band at around ∼3500 cm^–1^, which is associated with the O–H stretching vibrations. This broad band indicates moisture absorption, correlating with the hydrophilic nature of the VPVCap polymer (Figure 8).^52^ Furthermore, most of the characteristic bands observed in the FTIR spectra of the SPIONs-VTMS-VPVCap samples align with those of the polymer, confirming the successful incorporation of the polymer onto the nanoparticle surface. These results provide evidence of the functionalization of SPIONs with both VTMS and the further rafting of VPVCap.

**Figure 8:**
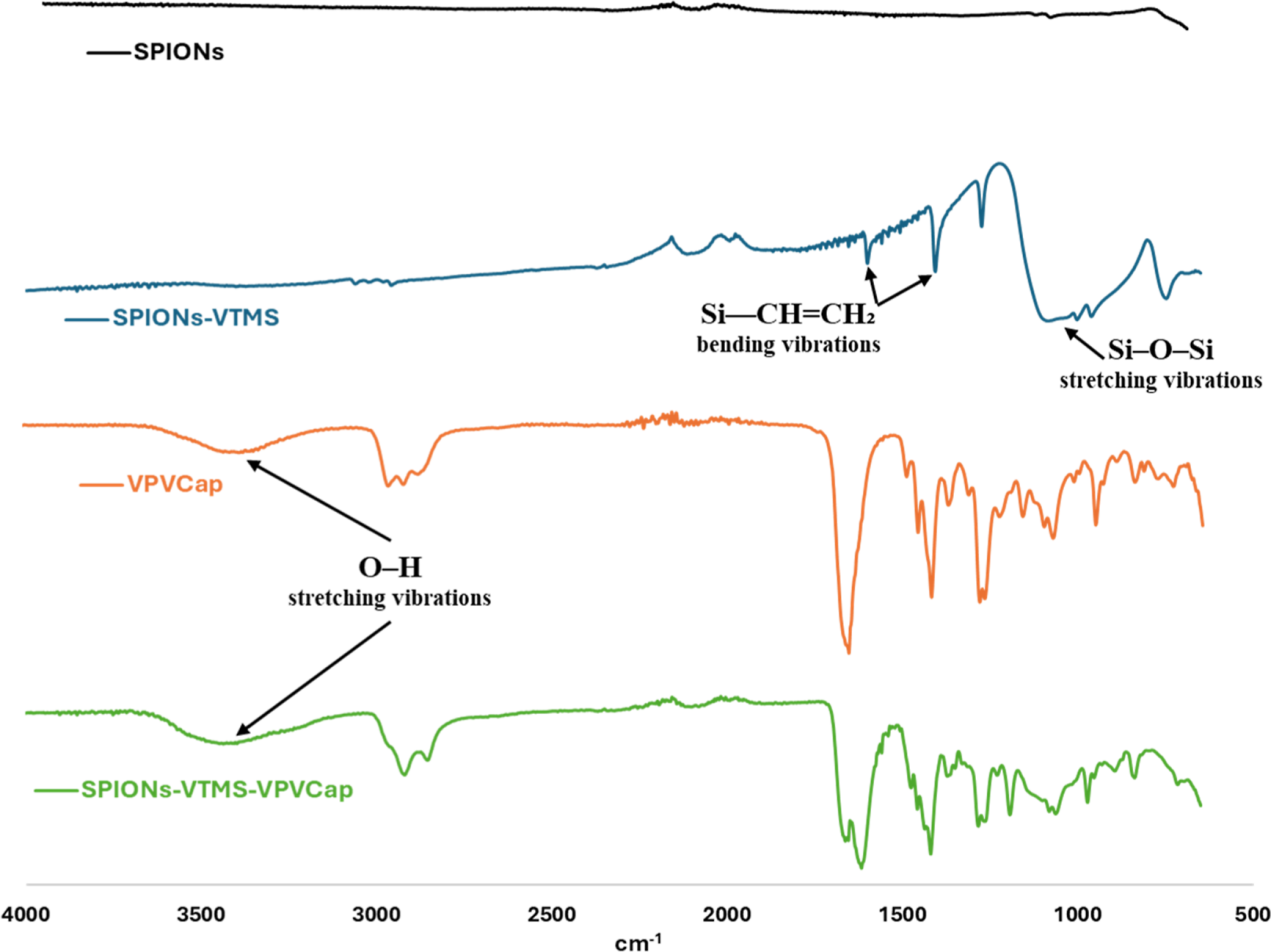
FTRI spectra of synthesized SPIONs, SPIONs-VTMS, VPVCap polymer, and SPIONs-VTMS-VPVCap.

### Zero Marine Environmental Test

#### Nuclear Magnetic Resonance

Achieving zero marine discharge of hydrate inhibitors is one of the primary goals when SPIONs-VTMS-VPVCap KHIs are applied offshore. Following the application of our smart nanocomposite-based SI using the SCC hydrate test, the nanocomposite particles were collected by a magnet to minimize environmental discharge. To claim zero environmental waste, ensuring no polymeric leakage after application is essential. Therefore, after the SCC kinetic hydrate inhibition tests, a 5000 ppm sample of SPIONs-VTMS-VPVCap was heated to 80 °C, which was the cloud point, and pH 12 and collected by a magnet, as shown in Figure 9, and two drops of the remaining solution were analyzed by ^1^H NMR spectroscopy. The analyses confirmed the absence of organic residues in the solution post-test and after recollecting the SPIONs-VTMS-VPVCap nanocomposite compared to the original ^1^H NMR for the copolymer VTMS-VPVCap, as shown in Figure 10.

**Figure 9:**
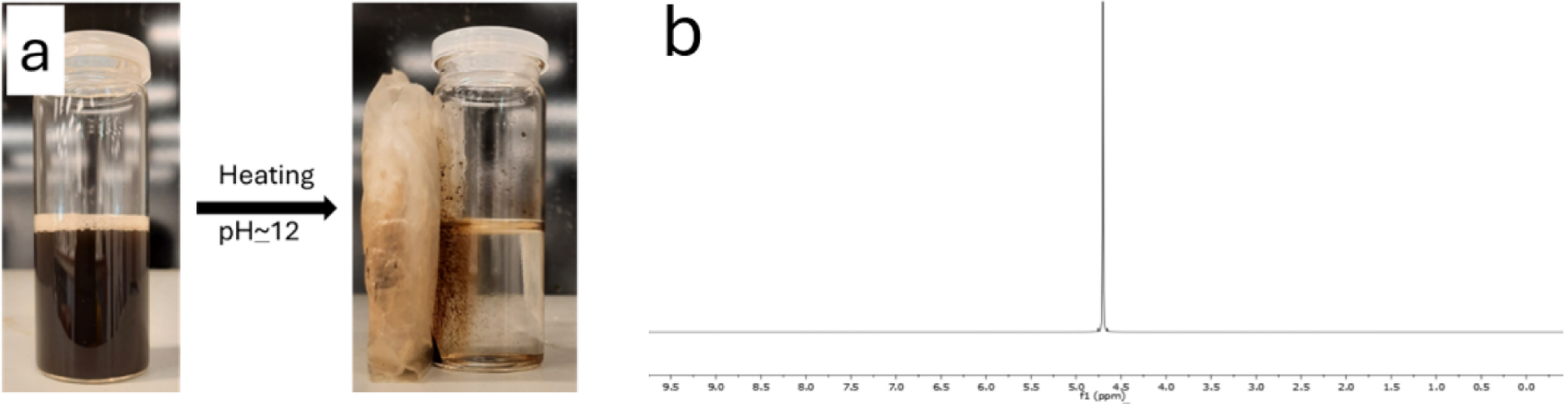
Heat treatment of aqueous SPIONs-VTMS-VPVCap: (a) sample before heating and (b) sample after heating with ^1^H NMR spectrum of the aqueous solution after magnetic recovery of SPIONs-VTMS-VPVCap.

**Figure 10:**
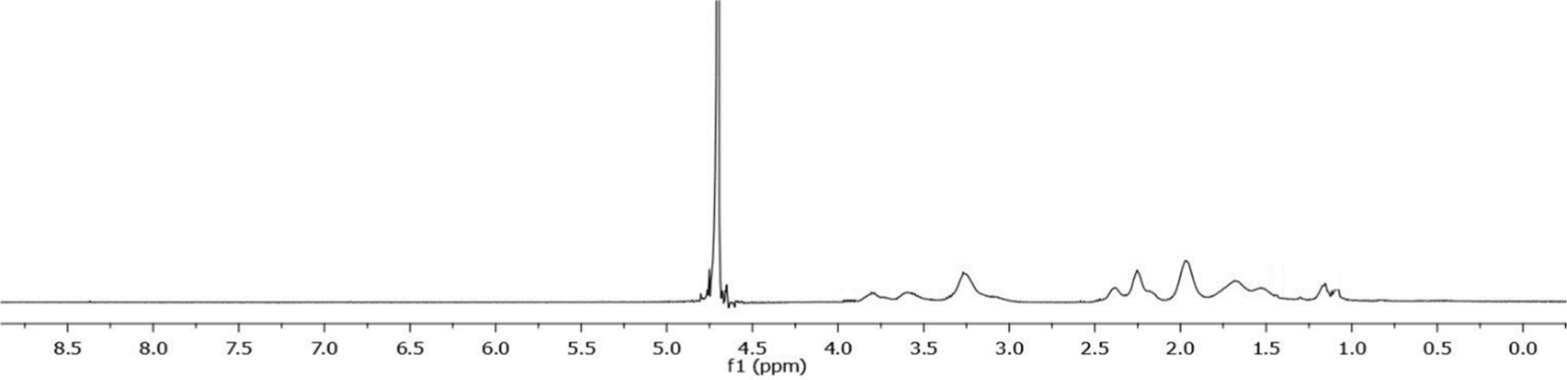
^1^H NMR of the copolymer VTMS-VPVCap.

#### Magnetization Measurement

The magnetic measurement results of the synthesized SPIONs and their functionalized derivatives (SPIONs-VTMS and SPIONs-VTMS-VPVCap) revealed significant differences in magnetization properties due to successive coatings. The key magnetization characteristics, namely, saturation magnetization (Ms), coercivity field (Hc), and remnant magnetization (Mr), were obtained from the hysteresis loops and are summarized and shown in Table 1 and Figure 11.

**Table 1:** Magnetic Properties of SPIONs, SPIONs-VTMS, and SPIONs-VTMS-VPVCap

	SPIONs	SPIONs-VTMS	SPIONs-VTMS-VPVCap
Ms (emu/g)	67	40	5.6
Hc (Oe)	5.9	4.5	2.2
*M*_r_ (emu/g)	0.54	0.32	0.03

**Figure 11:**
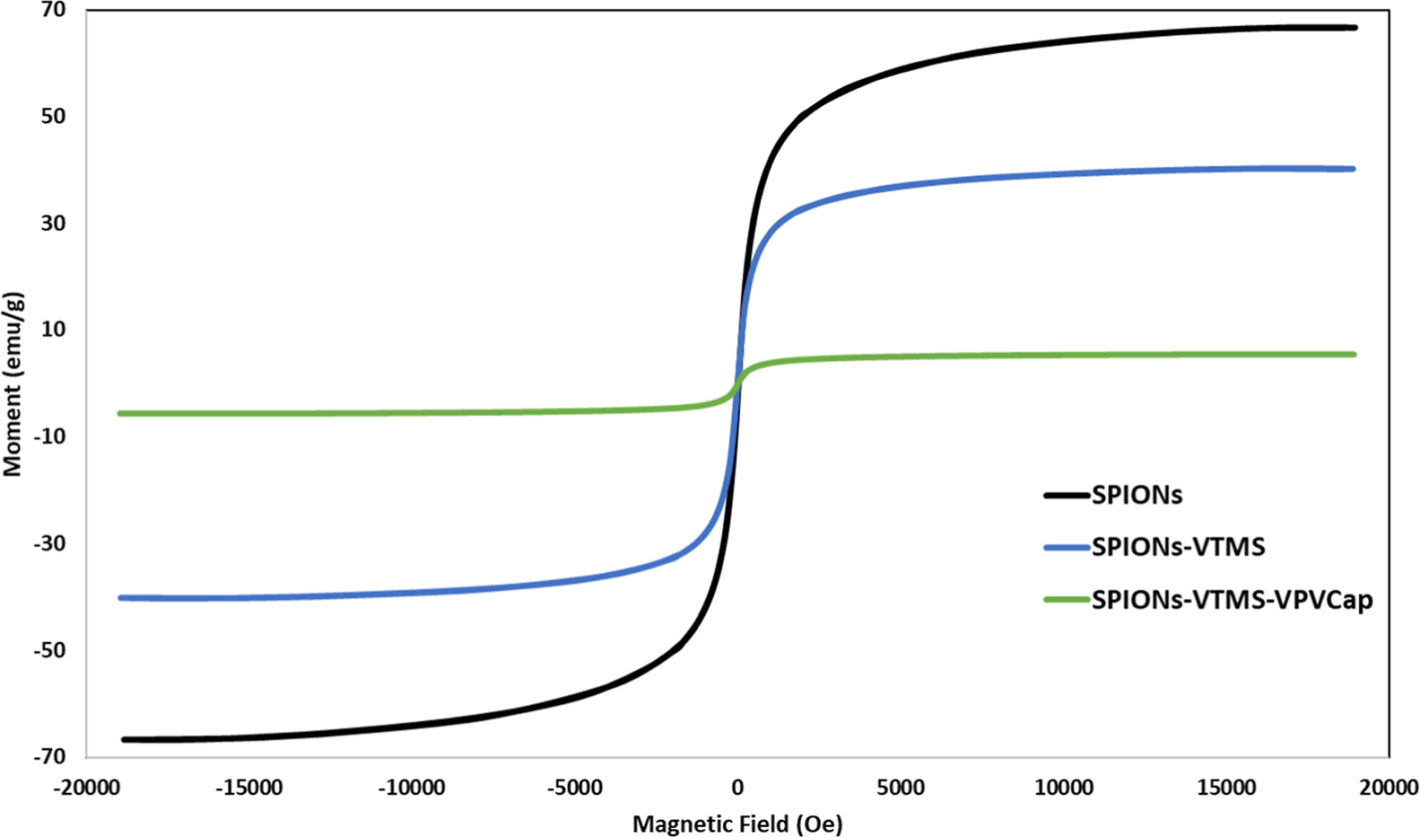
VSM hysteresis loops of SPIONs, SPIONs-VTMS, and SPIONs-VTMS-VPVCap.

The *M*_s_ value represents the maximum magnetization of the material under an applied magnetic field, while *H*_c_ is the magnetic field strength required to demagnetize the material (magnetic field required to return to zero magnetization state), and *M*_r_ is the residual magnetization at zero field (value of magnetization at zero field). The results demonstrate that the synthesized SPIONs exhibited a high *M*_s_ value of 67 emu/g, indicating a strong magnetization capacity. In contrast, functionalization with VTMS and further coating with VPVCap gradually decreased *M*_s_ to 40 and 5.6 emu/g, respectively. This reduction in magnetization is attributed to the nonmagnetic nature of the coating layers, which act as magnetic insulating barriers without compromising the recyclability of the nanoparticles.^16^

Similarly, the Hc values decreased from 5.9 to 4.5 Oe for SPIONs-VTMS and 2.2 Oe for SPIONs-VTMS-VPVCap. The low *H*_c_ values across all samples suggest their soft magnetic behavior with individual particles, forming single magnetic domains. This feature is crucial for ensuring the dispersibility of the nanoparticles in aqueous media and preventing aggregation in the absence of a magnetic field. Furthermore, the *M*_r_ values showed a significant reduction from 0.54 emu/g in SPIONs to 0.32 emu/g in SPIONs-VTMS and 0.03 emu/g in SPIONs-VTMS-VPVCap, confirming that the coating layers enhance the ability of SPIONs to act as a single magnetic domain and further confirm the successful surface functionalization and coating.

These results highlight the interplay between the magnetic properties and surface modifications of SPIONs, confirming the successful coating process while maintaining the magnetic functionality needed for our application.

#### Stability Observations

The stability of SPIONs-VTMS-VPVCap in both deionized (DI) water and brine (0.5 wt % NaCl) was evaluated by observing their dispersion over time, as shown in Figure 12. The samples were prepared at a concentration of 5000 ppm and stored under ambient conditions. Images were captured after 24 h (Figure 12a) and 1 week (Figure 12b) to assess sedimentation and agglomeration.

**Figure 12:**
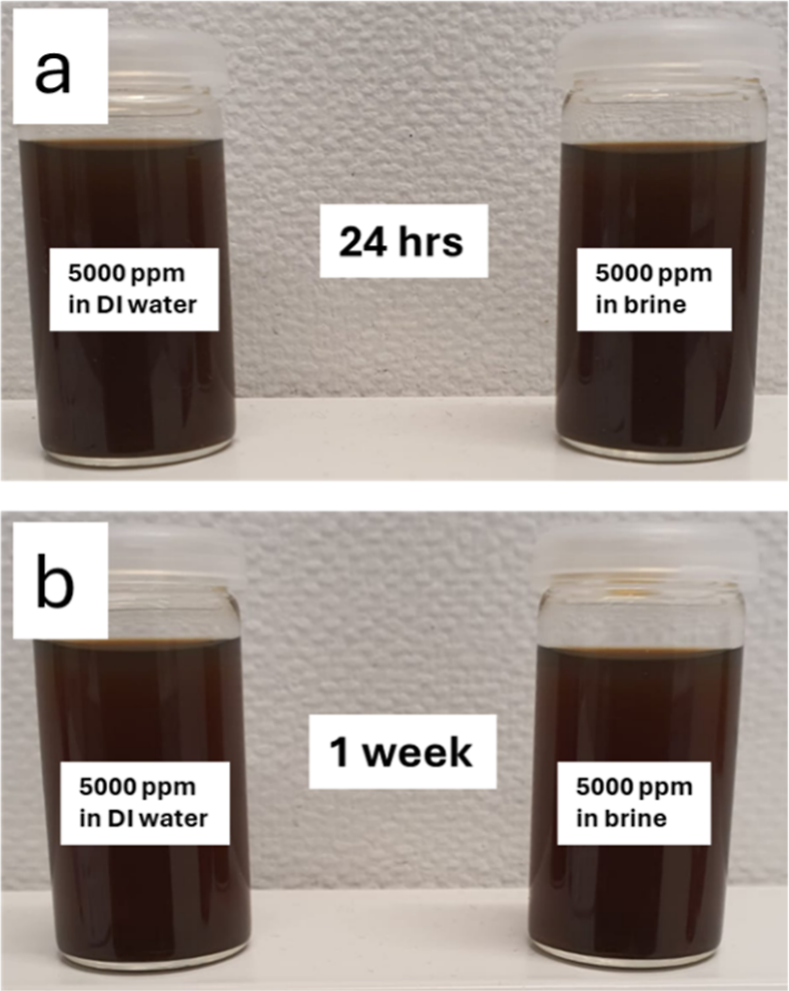
Stability observation of 5000 ppm of SPIONs-VTMS-VPVCap in DI water and 0.5 wt % NaCl brine solution after (a) 24 h and (b) 1 week.

In DI water, the SPIONs-VTMS-VPVCap remained well-dispersed, with no visible sedimentation or phase separation observed even after 1 week. This indicates excellent colloidal stability, likely due to the steric and electrostatic repulsion imparted by the surface coatings. In brine, the stability of SPIONs-VTMS-VPVCap was similarly robust, with no significant sedimentation or aggregation detected after 1 week. The presence of 0.5 wt % NaCl typically challenges nanoparticle stability due to potential screening of surface charges and salt-induced aggregation. However, the functionalization with VPVCap appears to provide sufficient steric hindrance and stability, ensuring dispersion even in saline environments.^16^

These results demonstrate the high stability of SPIONs-VTMS-VPVCap under both aqueous and saline conditions, making them suitable for hydrate inhibition applications.

### KHI Test Results

Table 2 summarizes the KHI performance results using the SCC test method and SNG as the gas phase in the steel rocking cells. We observed the first pressure drop due to hydrate formation at an average *T*_o_ of 17.0 °C. To compare the SPIONs-VTMS-VPVCap nanoparticles to pure polymer, we tested the VP/VCap copolymer synthesized from the VP and VCap monomers in the presence of VTMS. The copolymer was tested at 4000 ppm since this was the same active weight concentration of polymer when using 5000 ppm of SPIONs-VTMS-VPVCap, which has 80 wt % polymer. The VP/VCap copolymer gave an average *T*_o_ of 11.4 °C. The polymer molecular weight was not quantified, but from previous studies, we typically obtain *M*_n_ (number-average molecular weight) values in the range 6000–8000 g/mol.^53^

**Table 2:** Slow Constant Cooling KHI Test Results^a^

composite	concentration [ppm]	synergist and concentration	Av. *T*_o_ °C	Av. *T*_a_ °C	Av. *T*_o_–*T*_a_ °C
no additive			17.0	16.8	0.2
VPVCap copolymer	4000		11.4	8.8	2.6
SPIONs-VTMS-VPVCap	5000		12.9	12.4	0.6
SPIONs-VTMS-VPVCap first recycle	5000		12.0	10.5	1.5
SPIONs-VTMS-VPVCap second recycle	5000		12.4	10.3	2.2
SPIONs-VTMS-VPVCap third recycle	5000		12.5	11.3	1.3
SPIONs-VTMS-VPVCap fourth recycle	5000		11.7	11.0	0.7
SPIONs-VTMS-VPVCap	5000	BGE 5000 ppm	7.3	5.5	1.8

a The *T*_o_ values are the average of five tests.

When tested at 5000 ppm, the fresh SPIONs-VTMS-VPVCap gave an average *T*_o_ of 12.9 °C. This was statistically a little worse than the pure copolymer but still showed good KHI activity. Interestingly, when the nanoparticles were recovered using a magnet from the liquid in the cells and fresh 5000 ppm solutions made with the same nanoparticles, the KHI performance was not diminished. KHI tests with the first, second, and third recycled material gave average *T*_o_ values of 12.0, 12.4, and 12.4 °C. These values were lower than the original solution but not statistically significant (*p* > 0.05). However, the material recovered and used in the fourth recycle gave a significantly lower average *T*_o_ value of 11.7 °C. These results clearly demonstrate that the nanoparticles have not lost their KHI activity and may have begun to show some increased KHI activity, a reason for which we are not able to explain.

A possible reason for the lower KHI performance of SPIONs-VTMS-VPVCap compared to the VPVCap copolymer is related to the product structure. The polymer chains in SPIONs-VTMS-VPVCap radiate out from the central magnetic nanoparticle, much like a dendrimer or hyperbranched polymer, several classes of which are known to function as KHIs such as poly­(ester amide)­s.^3,21^ However, the hyperbranched KHIs are observed to have somewhat lower performance than single chain KHI polymers.^23^ This is presumed to be because the chains in the dendrimer are close and will double up unnecessarily in their interactions with the bulk water or gas hydrate particle surfaces. The SPIONs-VTMS-VPVCap will have a similar disadvantage, which will lead to a somewhat diminished performance (higher *T*_o_ values) compared to the VPVCap copolymer.

We also carried out tests with an added BGE solvent synergist. This solvent is used in several commercial KHI formulations and is biodegradable.^54^ 5000 ppm of SPIONs-VTMS-VPVCap with added 5000 ppm BGE gave excellent synergy while still keeping the nanoparticles suspended and monodisperse. The added BGE lowered the average *T*_o_ value to 7.3 °C, more than 5 °C lower than those in any of the tests with SPIONs-VTMS-VPVCap alone. The average *T*_o_ value of 7.3 °C was 9.7 °C better than no additive and approximately 13 °C lower than the hydrate equilibrium temperature at the pressure of the hydrate onset.

## Conclusion

Monodisperse superparamagnetic nanoparticles coated with over 80 wt % active *N*-vinylpyrrolidone/*N*-vinyl caprolactam (VP/VCap) copolymer chains (SPIONs-VTMS-VPVCap) were synthesized. These nanoparticles’ average was shown by DLS to have an average particle size of approximately 205 nm. 5000 ppm solutions of SPIONs-VTMS-VPVCap remained stable for several days, which is critical for shut-in scenarios as the KHI must remain distributed throughout the whole aqueous phase in order to prevent hydrate formation. Aqueous SPIONs-VTMS-VPVCap solutions gave a cloud point of 80 °C, similar to that of free VP/VCap copolymer, demonstrating their applicability for injection into fluids at high wellhead temperatures.

High-pressure rocking cell tests with a synthetic natural gas mixture and a constant cooling rate of 1 °C/h were carried out. A 5000 ppm aqueous solution of SPIONs-VTMS-VPVCap demonstrated good KHI performance. The average onset temperature for hydrate formation was 12.9 °C, only 1.5 °C higher than free VP/VCap copolymer at an equivalent wt % concentration. Furthermore, the SPIONs-VTMS-VPVCap nanoparticles could be recovered in quantitative yield and recycled many times without a loss of performance. The SPIONs-VTMS-VPVCap nanoparticles were also compatible with the solvent synergist, *n*-butyl glycol ether (BGE). Addition of 5000 ppm BGE to 5000 ppm of SPIONs-VTMS-VPVCap gave an average *T*_o_ value of 7.3 °C, which is 9.7 °C lower than the value obtained with no additive.

We believe this is the first study in which magnetic nanoparticle KHIs have ticked all of the boxes for proof of concept. This includes cheap, facile, and high-yield synthesis, monodisperse solutions with long-term stability without settling, high cloud point, compatibility with high flash point solvent boosting the KHI performance, and proof that the recycled nanoparticles do not lose efficacy. These results will help move the industry toward the goal of zero chemical discharge to the marine environment. We are evaluating the economics of using magnetic nanoparticle KHIs against the current commercial KHIs and hope to report on this in the future.
